# Research Note: Comparison of two methods to measure broiler tibia morphology

**DOI:** 10.1016/j.psj.2022.102245

**Published:** 2022-10-13

**Authors:** A. Magnaterra, R. Mitchell, C.R. Angel, M. Khong, Z. McMillian, A. Snyder, S. Weimer

**Affiliations:** ⁎ Department of Animal and Avian Science, University of Maryland, College Park, MD 20742, USA; † Perdue Foods, LLC, Salisbury, MD 21801, USA; ‡ Department of Poultry Science, University of Arkansas, Fayetteville, AR 72701, USA

**Keywords:** broiler, morphology, tibia, methodology, digital image

## Abstract

The skeletal integrity of chickens is an important area of research and detailed measures are needed to better understand the influence of experimental manipulation on bone health. The objective of this experiment was to compare 2 methods to measure the superficial tibiotarsus (tibia) morphology of broiler chickens collected in the wet laboratory (**WL**) or from digital images (**DIG**). The length, width at 90%, 75%, 50%, 25%, and 10% of the length, proximal and distal head width, medial, lateral, and distal intercondylar groove depth (**ID**), and proximal head angle were measured on the right and left tibias collected from broilers in 2 experiments (E1, E2). In both experiments, tibias had a greater width at 90% of the length when measured with the WL method compared with the DIG method (*P* ≤ 0.04), while tibias measured with the DIG method had a greater length, distal ID, and widths at 10%, 25%, 50%, and 75%, of the length compared with the WL method (*P* < 0.0001). In E1, tibias measured with the DIG method had a greater medial, lateral, and distal ID compared with the WL method (*P* ≤ 0.04). In E2, compared with the DIG method, tibias measured with the WL method had a greater distal head width and lateral ID, yet a shallower distal ID (*P* ≤ 0.03). The use of the DIG method provided more precise measures but, due to the limitations of measures from digital images and the opportunity for more accurate measures to be collected with the WL method, the WL method is recommended to measure the superficial morphology of broiler chickens because it was more accessible and practical.

## INTRODUCTION

Broiler chickens have increased in body weight and growth rate over the past 60 years and there is concern that skeletal growth may not occur at the same rate as muscle growth (Havenstein et al., 2003; Shim et al., 2012). Due to the prevalence of these issues, there is a need for distinctive measures of bone integrity which provide detailed insight into the health status of the broiler tibiotarsus (referred to as tibia) in the laboratory environment. Measures of skeletal integrity and health can include bone breaking strength, mineralization (ash content), cortical thickness and porosity, and bone morphology (Dilworth and Day, 1965; Black and Mattson, 1982; Cruickshank and Sim 1986; Rath et al., 1999; Bonser and Casinos, 2003). Bone breaking strength is influenced by both the organic matrix and inorganic content (ash) present in the bone (Rath et al., 1999) and is commonly measured by placing mechanical pressure at 3 points along the bone to break at the middle (ASABE, 1992), limiting measures of bone strength to the midpoint (mid diaphysis) of the bone. Multiple morphology measures at precise locations along the bone could provide more detail about integrity of the tibia.

Previous research has been conducted to understand the effects of breed, age, nutrition, and environment on the tibia morphology of broilers (Cruickshank and Sim, 1986; Shim et al., 2012; Guo et al., 2019; Sanchez-Rodriguez et al., 2019; Harash et al., 2020; Pedersen et al., 2020). Measures of superficial tibia morphology have included length (Cruickshank and Sim, 1986; Shim et al., 2012; Guo et al., 2019; Sanchez-Rodriguez et al., 2019; Harash et al., 2020; Pedersen et al., 2020), width of the mid diaphysis (Cruickshank and Sim, 1986; Shim et al., 2012; Guo et al., 2019; Harash et al., 2020; Pedersen et al., 2020), proximal head (Guo et al., 2019), and distal condyle (Cruickshank and Sim, 1986), as well as depth of the distal intercondylar groove (Cruickshank and Sim, 1986). These morphology measurements have been performed either in a laboratory using calipers (Cruickshank and Sim, 1986; Pedersen et al., 2020) or with imaging technology (Cruickshank and Sim, 1986; Sanchez-Rodriguez et al., 2019; Harash et al., 2020), but not both. Radiograph images (Cruickshank and Sim, 1986) and computed tomography (**CT**) scans (Harash et al., 2020) have also been used and, while these technologies have been proven to be effective for more in-depth analysis of bone morphology, access to CT and radiograph technologies can be limited due to cost. However, the cost of imaging using a digital camera is significantly lower. If morphology measures along the length of the tibia could be taken using images from a digital camera, detailed analysis of bone morphology could be more accessible to researchers with limited resources.

The objective of this experiment was to compare two different methods of measuring superficial tibiotarsus (referred to as tibia) morphology of broiler chickens in two experiments. Comparison of tibia morphology measures collected in the wet laboratory (**WL**) to measures collected with Fiji ImageJ Software (Schindelin et al., 2012) from digital images taken with a DSLR camera (**DIG**) was done to evaluate differences in methodology.

## MATERIALS AND METHODS

For experiment 1 (**E1**), the left and right tibia bones were collected from 120 commercial broiler chickens at 51 d of age and boiled in water to remove the flesh and articular cartilage and shipped to the University of Maryland. For experiment 2 (**E2**), the left and right tibia bones were collected from 85 commercial broiler chickens at 53 d of age by manually defleshing and removing articular cartilage from the fresh bone (Li et al., 2015). Each tibia was assigned a tag number and stored at −20°C. All animal procedures were approved by the University of Maryland Institutional Animal Care and Use Committee (protocol R-JUL-20-35).

### Tibia Superficial Morphology––Wet laboratory

Wet laboratory (**WL**) measures were performed on thawed and air dried tibia bones using a digital caliper (iGaging IP54, San Clemente, CA) to collect the length (mm), proximal head width (**PHW**; mm), distal head width (**DHW**), width at 10%, 25%, 50%, 75%, and 90% of the length (starting from the distal head), depth (mm) of the proximal head medial (**MID**) and lateral (**LID**) intercondylar grooves, and depth (mm) of the distal head intercondylar groove (**DID**) (E1 N = 240 tibias; E2 N = 170 tibias). The total tibia length was used to calculate the lengths at which widths were measured at 90%, 75%, 50%, 25%, and 10% of the total length. The angle of the proximal head through the medial to lateral condyle was measured using a protractor.

### Tibia Superficial Morphology––Digital

Digital image (**DIG**) morphological measures were recorded for the same right and left tibia bones (E1 N = 240 tibias, E2 N = 170 tibias) as the WL method. Tibias were individually placed on a 1 cm grid printed on yellow paper in a light box with an orange background during imaging. Images of the anterior, proximal, and distal surfaces of each tibia were taken using a digital single lens reflex (**DSLR**) camera (a6400, Sony, Minato City, Tokyo, Japan) that was mounted to a tripod and affixed with a macro lens (E 30 mm f/3.5 macro lens, Sony, Minato City, Tokyo, Japan). The camera was set to manual mode to ensure the same exposure time and aperture were used for all images. The ISO was set to 100 to optimize the resolution of the images. The exposure time was 1/8 s, and the F-stop was set to 22 to maximize depth of field. The direct manual focus setting was used. A point-and-shoot digital camera was also tested, but did not provide the same control settings, and was not used for final images used in analysis. A focal length of 18 cm was used for the anterior surface images and a 12 cm focal distance was used for the proximal and distal images.

**Figure 1:**
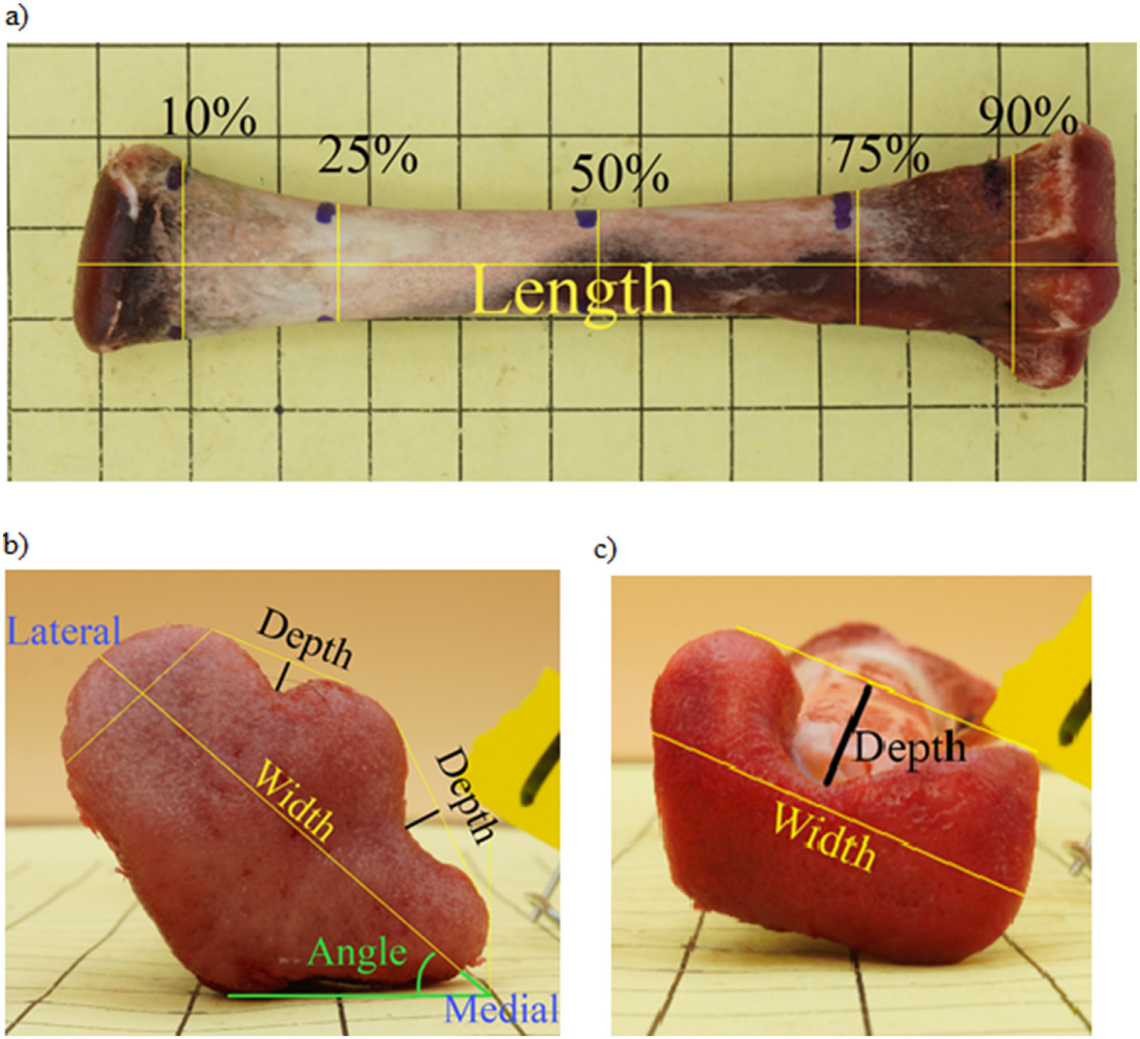
Morphology measures collected on digital images of right and left broiler chicken tibias consisted of (A) length and widths at 10%, 25%, 50%, 75%, 90% of the length, (B) proximal head angle (green), width (yellow), and lateral and medial intercondylar depths (black) and (C) distal head width (yellow) and intercondylar depth (black).

DIG morphology measurements were collected using Fiji ImageJ software (National Institute of Health, Bethesda, Maryland; Schindelin et al., 2012). A 1 cm line from the grid paper in each image was used to calibrate the scale as a ratio of pixels to millimeters (pixel: mm). The tibia length (mm) and width (mm) at 10%, 25%, 50%, 75%, and 90% of the length were collected from the anterior image. The fixed length line tool macro (Schmid and Rasband, 2015) was used to mark the width locations for each length percentage. The widths were measured using the line tool (Figure 1A). The depth of the MID and LID were measured on the proximal image by drawing a line across the most anterior portion of the condyles of the proximal head to represent the highest point (Figure 1B) and the DID was measured on the distal image by drawing a line across the most distal portion of the condyle of the distal head to represent the highest point (Figure 1C). Next, a line was drawn perpendicular to the first line at the deepest point of the intercondylar groove. The line tool was used to measure the width of the proximal head in the proximal image (Figure 1B) and the width of the distal head in the distal image (Figure 1C). The angle tool was used to measure the angle of the proximal head through the medial to lateral condyle and by drawing a line from the top of the anterior surface of the lateral condyle to the posterior surface of the bone. The midpoint of the first line was used as a reference through which the angle was drawn (Figure 1B).

### Statistical Analysis

Left and right tibias were measured separately and averaged for analysis for both WL and DIG image methods. Statistical analysis was completed using JMP Pro 14 software (SAS Institute Inc. Cary, NC). Data was analyzed using separate t-tests to compare the morphology measures across the two methods (WL or DIG image) within each experiment and to compare the two methods of morphology measures across the two experiments (E1 and E2) within each method. For all measures, a *P*-value of *P* ≤ 0.05 was considered significant and a trend at *P* ≤ 0.10.

## RESULTS AND DISCUSSION

Results are presented in Table 1. In E1 and E2, tibia length and widths at 10%, 25%, 50%, and 75% of the length were greater (*P* < 0.0001) when measured with the DIG method than with the WL method. These differences were potentially due to the calibration of the pixel:mm scale set for DIG image measures. The measures were collected in mm, but the scale was set using a 1 cm line to be equal to 10 mm, which may have reduced measure accuracy. To further increase the accuracy of measures in the future, a 1 mm line should be used to calibrate the measurement scale instead of a 1 cm line. The width at 90% length was greater (*P* ≤ 0.04) for the WL method measures than DIG in both experiments. This was likely because of the torsion of the bone near the proximal head, which was not visible at widest point of the bone (medial to lateral) in some of the anterior surface images, but in the WL the bone was visually inspected to be measured at the widest point at each location.

**Table 1:** Comparison of left, right, and average (of the left and right) tibia length (mm), proximal head width (mm) and angle (°), distal head width (mm), width (mm) at 10%, 25%, 50%, 75%, and 90% of the length, and medial, lateral, and distal intercondylar groove depths (mm) measured in the wet laboratory (WL) and in digital images (DIG) from broilers in experiment 1 (E1; N = 240 tibias) and experiment 2 (E2; N = 170 tibias).

		Average			Left			Right		
Measure	Method	E1	E2	*P* value	E1	E2	*P* value	E1	E2	*P* value
Length	WL^1^	107.7 ± 0.5^b^^z^	113.9 ± 0.6^b^^,^^y^	<0.0001	107.7 ± 5.5^b^^z^	113.8 ± 4.3^b^^,^^y^	<0.0001	107.8 ± 5.4^b^^z^	113.9 ± 4.4^b^^,^^y^	<0.0001
	DIG^2^	118.1 ± 0.5^a^^z^	123.8 ± 0.5^a^^,^^y^	<0.0001	117.7 ± 6.5^a^^z^	123.7 ± 6.2^a^^,^^y^	<0.0001	118.4 ± 6.2^a^^z^	124.0 ± 5.5^a^^,^^y^	<0.0001
	*P* value	<0.0001	<0.0001		<0.0001	<0.0001		<0.0001	<0.0001	
PHW^3^	WL	28.7 ± 0.2^b^^z^	30.6 ± 0.2^y^	<0.0001	28.6 ± 0.2^z^	30.6 ± 0.2^y^	<0.0001	28.7 ± 0.2^b^^z^	30.6 ± 0.2^y^	<0.0001
	DIG	29.1 ± 0.2^a^^z^	30.8 ± 0.2^y^	<0.0001	29.1 ± 0.2^z^	30.7 ± 0.2^y^	<0.0001	29.2 ± 0.2^a^^z^	30.8 ± 0.2^y^	<0.0001
	*P* value	0.04	0.64		0.08	0.64		0.05	0.41	
Angle	WL	35.1 ± 0.4^b^^z^	37.5 ± 0.3^b^^,^^y^	<0.0001	34.8 ± 0.5^b^^z^	37.3 ± 0.4^b^^,^^y^	<0.0001	35.4 ± 0.5^b^^z^	37.7 ± 0.4^y^	0.0003
	DIG	39.8 ± 0.3^a^^,^^y^	38.8 ± 0.4^a^^z^	0.02	39.5 ± 0.3^a^	39.2 ± 0.4^a^	0.45	40.2 ± 0.4^a^^,^^y^	38.4 ± 0.4^z^	0.001
	*P* value	<0.0001	0.007		<0.0001	0.0005		<0.0001	0.19	
DHW	WL	22.1 ± 0.1^z^	22.8 ± 0.2^a^^,^^y^	0.0004	22.2 ± 0.1^z^	22.9 ± 0.2^a^^,^^y^	0.0008	22.0 ± 0.1^z^	22.7 ± 0.2^a^^,^^y^	0.0004
	DIG	22.0 ± 0.1	22.2 ± 0.2^b^	0.35	22.0 ± 0.1	22.1 ± 0.2^b^	0.64	21.9 ± 0.1	22.2 ± 0.2^b^	0.25
	*P* value	0.35	0.009		0.19	0.004		0.8	0.03	
90%	WL	24.7 ± 0.2^a^^z^	25.7 ± 0.2^a^^,^^y^	0.0007	24.7 ± 0.2^a^^z^	25.6 ± 0.2^a^^,^^y^	0.003	24.8 ± 0.2^a^^z^	25.8 ± 0.2^a^^,^^y^	0.002
	DIG	24.0 ± 0.2^b^^z^	24.7 ± 0.2^b^^,^^y^	0.03	23.9 ± 0.2^b^^z^	25.0 ± 0.3^b^^,^^y^	0.0009	24.1 ± 0.3^b^	24.4 ± 0.3^b^	0.43
	*P* value	0.009	0.003		0.02	0.04		0.04	0.0001	
75%	WL	14.1 ± 0.1^b^^z^	14.4 ± 0.1^b^^,^^y^	0.03	14.0 ± 0.1^b^	14.3 ± 0.1^b^	0.11	14.1 ± 0.1^b^^z^	14.5 ± 0.1^b^^,^^y^	0.04
	DIG	14.9 ± 0.1^a^^z^	15.5 ± 0.1^a^^,^^y^	0.002	14.8 ± 0.1^a^^z^	15.4 ± 0.1^a^^,^^y^	0.002	14.9 ± 0.1^a^^z^	15.5 ± 0.2^a^^,^^y^	0.004
	*P* value	<0.0001	<0.0001		<0.0001	<0.0001		<0.0001	<0.0001	
50%	WL	10.2 ± 0.08^b^^z^	11.1 ± 0.1^b^^,^^y^	<0.0001	10.2 ± 0.08^b^^z^	11.1 ± 0.1^b^^,^^y^	<0.0001	10.2 ± 0.08^b^^z^	11.0 ± 0.1^b^^,^^y^	<0.0001
	DIG	11.0 ± 0.08^a^^z^	11.8 ± 0.1^a^^,^^y^	<0.0001	11.0 ± 0.08^a^^z^	11.9 ± 0.1^a^^,^^y^	<0.0001	11.0 ± 0.08^a^^z^	11.8 ± 0.1^a^^,^^y^	<0.0001
	*P* value	<0.0001	<0.0001		<0.0001	<0.0001		<0.0001	<0.0001	
25%	WL	12.0 ± 0.07^b^^z^	12.3 ± 0.1^b^^,^^y^	0.003	12.0 ± 0.08^b^^z^	12.4 ± 0.1^b^^,^^y^	0.004	11.9 ± 0.07^b^^z^	12.3 ± 0.1^b^^,^^y^	0.003
	DIG	13.0 ± 0.08^a^^z^	13.4 ± 0.1^a^^,^^y^	0.02	13.0 ± 0.08^a^^z^	13.4 ± 0.1^a^^,^^y^	0.01	13.0 ± 0.08^a^^z^	13.4 ± 0.1^a^^,^^y^	0.01
	*P* value	<0.0001	<0.0001		<0.0001	<0.0001		<0.0001	<0.0001	
10%	WL	18.0 ± 0.1^b^	18.0 ± 0.1^b^	0.87	18.1 ± 0.1^b^	18.1 ± 0.1^b^	0.64	17.9 ± 0.1^b^	17.8 ± 0.2^b^	0.47
	DIG	21.0 ± 0.1^a^	20.5 ± 0.2^a^	0.08	21.0 ± 0.1^a^^,^^y^	20.5 ± 0.2^a^^z^	0.02	21.0 ± 0.2^a^	20.8 ± 0.2^a^	0.44
	*P* value	<0.0001	<0.0001		<0.0001	<0.0001		<0.0001	<0.0001	
MID	WL	1.6 ± 0.03^b^^z^	1.8 ± 0.03^y^	<0.0001	1.5 ± 0.04^b^^z^	1.8 ± 0.03^y^	<0.0001	1.6 ± 0.04^b^^z^	1.8 ± 0.05^y^	0.02
	DIG	1.7 ± 0.03^a^^z^	1.9 ± 0.03^y^	0.0003	1.7 ± 0.04^a^^z^	1.9 ± 0.04^y^	<0.0001	1.8 ± 0.03^a^^z^	1.9 ± 0.04^y^	0.0002
	*P* value	0.0001	0.23		0.04	0.23		0.0008	0.25	
LID	WL	1.7 ± 0.03^b^^z^	2.2 ± 0.04^a^^,^^y^	<0.0001	1.7 ± 0.04^b^^z^	2.2 ± 0.04^a^^,^^y^	<0.0001	1.7 ± 0.04^b^^z^	2.2 ± 0.05^a^^,^^y^	<0.0001
	DIG	1.9 ± 0.04^a^	1.9 ± 0.04^b^	0.97	1.9 ± 0.05^a^	1.9 ± 0.04^b^	0.93	1.9 ± 0.05^a^	1.9 ± 0.04^b^	0.87
	*P* value	<0.0001	<0.0001		0.0002	<0.0001		0.003	<0.0001	
DID	WL	5.8 ± 0.05^b^^z^	6.4 ± 0.06^b^^,^^y^	<0.0001	5.9 ± 0.06^b^^z^	6.4 ± 0.06^b^^,^^y^	<0.0001	5.7 ± 0.06^b^^z^	6.3 ± 0.06^b^^,^^y^	<0.0001
	DIG	6.6 ± 0.05^a^	6.6 ± 0.08^a^	0.63	6.5 ± 0.05^a^	6.6 ± 0.08^a^	0.61	6.6 ± 0.06^a^	6.6 ± 0.08^a^	0.74
	*P* value	<0.0001	0.007		<0.0001	0.03		<0.0001	0.003	

1 Wet lab (WL) method was performed using digital calipers on the tibias of broilers.

2 Digital method (DIG) was performed using ImageJ software on 2D images of the tibias of broilers.

3 Abbreviations: DHW, distal head width; DID, distal intercondylar groove depth; LID, lateral intercondylar groove depth; MID, medial intercondylar groove depth; PHW, Proximal head width.

ab Average, left, and right means not sharing the same letter within each experiment column indicates a significant difference across methods (WL vs. DIG).

xy Average, left, and right means not sharing the same letter across within each method row indicates a significant difference across experiments (E1 vs. E2).

In E1, average and right PHW were greater (*P* ≤ 0.05) for the DIG method, while the left tibia was trending (*P* = 0.08), compared with the WL method. Despite the differences at other width locations, the DHW was not different between the DIG or WL method in E1.

Additionally, E1 MID, LID, and DID were greater (*P* ≤ 0.04) for the DIG method than WL, and this was likely due to a limitation of the WL methods. This limitation was that the width of the digital caliper depth rod was sometimes wider than the width of the deepest part of the intercondylar groove. Finally, E1 proximal head angle was also greater (*P* <0.0001) for DIG than WL measures, likely a result of increased precision of the DIG methodology. In the WL measures, since tibias differed in torsion and curvature, the tibia did not always sit flat against the table surface and the researcher had to hold the tibia in position to measure the angle through the medial to lateral condyle. The DIG method used a reference angle that was adjusted for each tibia's torsion and curvature to go through the medial to lateral notch during measurement.

In E2, there was no effect of method on PHW and MID measures. The LID was greater (*P* < 0.0001) for the WL than DID measures. The torsion and intercondylar groove depth of the tibias in E1 did not present the same WL measure issues as E2, due to natural tibia morphology differences. For example, the depth rod was able to reach the deepest part of the intercondylar groove. The methodological difference in the intercondylar groove depths is likely because the DIG method can only measure the most proximal surface of the tibia image, but the depth may vary across the intercondylar groove, which can be captured in the WL method. The E2 tibia DHW was greater (*P* ≤ 0.03) with the WL method than DIG and this was likely because the location of the DHW measure was easier to standardize in the DIG than WL method but may not have been measured at the widest point. While the WL measures were more accurate, the DIG measures were more precise.

As expected, the overall length, and most widths, were greater for the tibias from the broilers that were 2 d older in E2 than in E1. The width at 10% length measure was consistently an exception, and the lack of difference with the DIG measures has also been reported in a study with 6-wk-old broilers (Cruickshank and Sim, 1986). When comparing the remaining measures between the 2 experiments within the WL methods, tibia measures were greater (*P* ≤ 0.04) in E2 than E1, except for the left tibia width at 75% length.

Within the DIG method, the length, widths at 25%, 50%, and 75% length, PHW, and MID were greater (*P* ≤ 0.05) in E2 than E1. Contrasting E1, the E2 average and left DIG tibia width at 90% length measures were greater (*P* ≤ 0.03) than WL measures, but this was not the case for the right tibia (*P* = 0.43) in E2. Within the DIG method, there was no effect of experiment on DHW measures and the left tibia width at 10% length was greater in E1 than E2 (*P* = 0.02), and this effect was a tendency for the average of both tibias (*P* = 0.08).

There was no difference in the LID and DID tibia measures collected with the DIG method across experiments. However, within the WL method, MID, LID, and DID measures were greater (*P* ≤ 0.0001) in E2 compared with E1. The potential source of these differences could be attributed to the caliper depth rod issues in the WL method as discussed previously the rod of the calipers used to collect the WL measures was often greater than the width of the intercondylar groove at the deepest point, preventing completely accurate measurements in E1.

Within the DIG method, proximal head angle measures did not differ across experiments for the left tibia, but the average and right tibia angles were greater in E1 than E2 (*P* ≤ 0.02). However, the WL method E2 tibia proximal head angle was greater than E1 (*P* ≤ 0.0003). This could be due to natural variation in the tibias between experiments, or due to variation in observers. There were 2 observers collecting data for WL methods in E1, but only one observer in E2. Notably, the angle measure could be the most subjective measure in the WL because the tibia may have to be held in position to measure the angle between the center of the medial to lateral condyles.

To summarize, the differences in the DIG and WL methods to measure the superficial morphology of tibia bones from broilers could at least partially be attributed to the greater accuracy of the WL method and greater precision of the DIG method. The fixed length line tool macro (Schmid and Rasband, 2015) used in the DIG method auto computed the precise location of the 10%, 25%, 50%, 75%, and 90% lengths to measure the widths of each bone, as opposed to the location being marked with a permanent marker by a researcher that allowed for the calipers to be placed at any point along the width of the mark in the WL method. Additionally, measures were limited to 2 decimal places with the digital calipers used in the WL, but DIG measures were out to 4 decimal places.

Despite the benefit of increased precision with the DIG method, there are still limitations. The superficial images were two-dimensional, but the bones are 3-dimensional in nature. Only one surface could be used in the 2-dimensional images, resulting in loss of detail. Any minor changes in bone morphology may have impacted how the bone rested on the surface on which it was photographed, affecting the image data. Additionally, the time spent recording measurements during the DIG method was greater than the WL method. To improve the accuracy of the DIG method, the ImageJ scale should be set using a line known to be 1 mm rather than using a 1 cm:10 mm scale. Both the loss of detail and the influence of individual bone morphology associated with the 2-dimensional images might be rectified with the use of a 3-dimensional model which could be rendered in a variety of software using modern technology. This 3-dimensional model could potentially then be measured using the auto calculated locations from the DIG measures, or other similar methods, for increased accuracy compared to the WL measures. Machine learning algorithms could be utilized to automate digital measures.

Both the wet laboratory and digital image methods can provide detailed understanding of morphology measures along the tibia bones of broilers, which may further the knowledge on bone health and integrity. Due to the limitations associated with the scale calibration and orientation of the bone in the anterior image in the digital methods, the wet laboratory methods to collect tibia length and width measures are recommended because this method was more accessible and practical, and the tibia can be manipulated in 3-dimensional space for more accurate measures.
